# Structures, functions, and syntheses of glycero-glycophospholipids

**DOI:** 10.3389/fchem.2024.1353688

**Published:** 2024-02-08

**Authors:** Tsukiho Osawa, Kohki Fujikawa, Keiko Shimamoto

**Affiliations:** ^1^ Bioorganic Research Institute, Suntory Foundation for Life Sciences, Kyoto, Japan; ^2^ Department of Chemistry, Graduate School of Science, Osaka University, Osaka, Japan

**Keywords:** glycero-glycophospholipid, phospholipid, glycolipid, glycerolipid, membrane lipid, membrane protein integration

## Abstract

Biological membranes consist of integral and peripheral protein-associated lipid bilayers. Although constituent lipids vary among cells, membrane lipids are mainly classified as phospholipids, glycolipids, and sterols. Phospholipids are further divided into glycerophospholipids and sphingophospholipids, whereas glycolipids are further classified as glyceroglycolipids and sphingoglycolipids. Both glycerophospholipids and glyceroglycolipids contain diacylglycerol as the common backbone, but their head groups differ. Most glycerolipids have polar head groups containing phosphate esters or sugar moieties. However, trace components termed glycero-glycophospholipids, each possessing both a phosphate ester and a sugar moiety, exist in membranes. Recently, the unique biological activities of glycero-glycophospholipids have attracted considerable attention. In this review, we describe the structure, distribution, function, biosynthesis, and chemical synthetic approaches of representative glycero-glycophospholipids—phosphatidylglucoside (PtdGlc) and enterobacterial common antigen (ECA). In addition, we introduce our recent studies on the rare glycero-glyco“pyrophospho”lipid, membrane protein integrase (MPIase), which is involved in protein translocation across biomembranes.

## 1 Introduction

**FIGURE 1:**
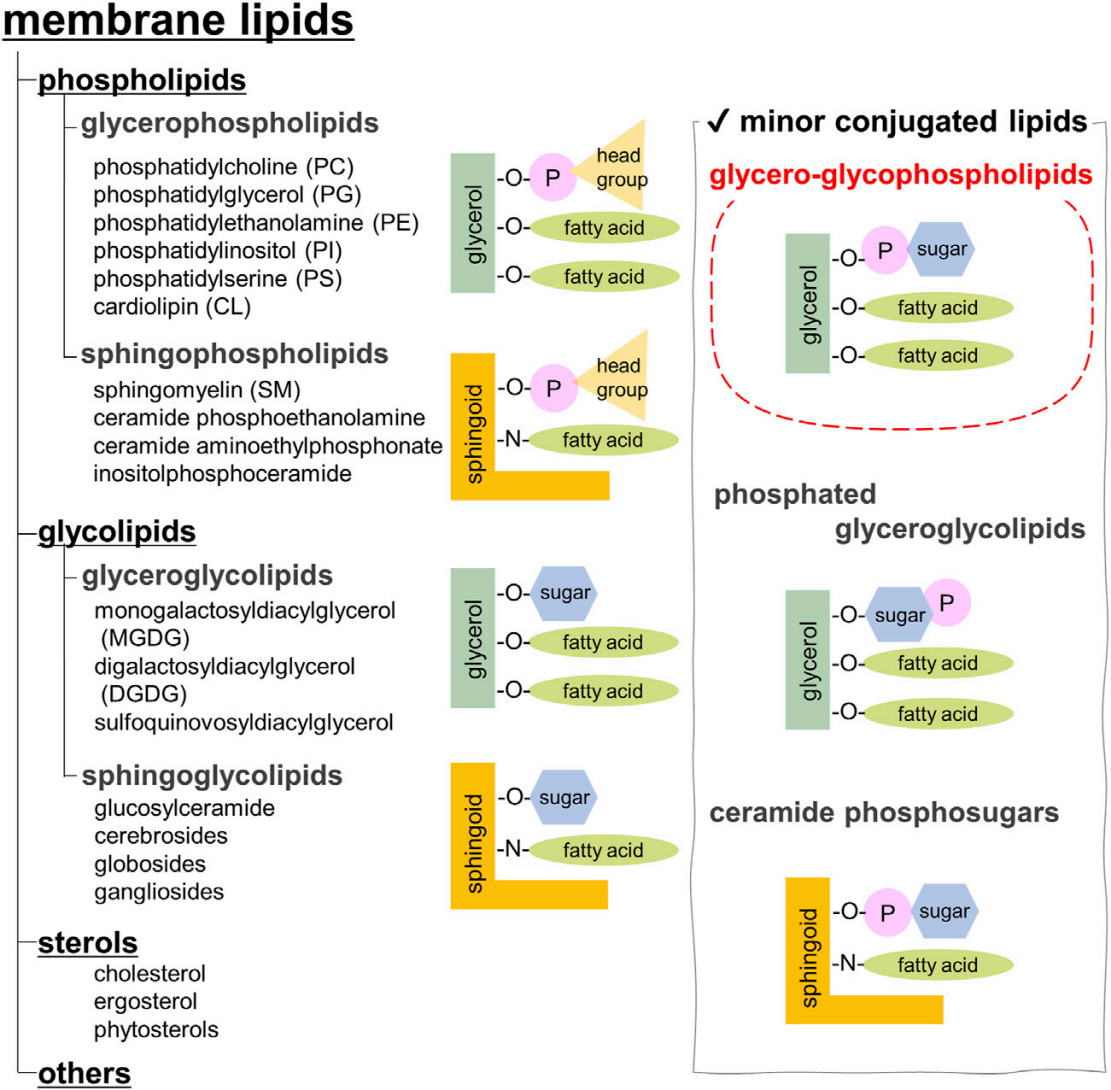
Classification of membrane lipids.

Biological membranes are vital for cell survival and consist of lipid bilayers associated with integral and peripheral proteins. The types of membrane lipids vary among species, tissues, and organelles, and are also known to be asymmetric in the outer and inner leaflets of a membrane (Escriba et al., 2015; Fujimoto and Parmryd, 2016). Membrane lipids are mainly classified as phospholipids, glycolipids, and sterols. Sterols are not found in the prokaryotic membranes. Animals, plants, and yeasts often contain cholesterol, phytosterols, and ergosterol, respectively (Figure 1).

**FIGURE 2:**
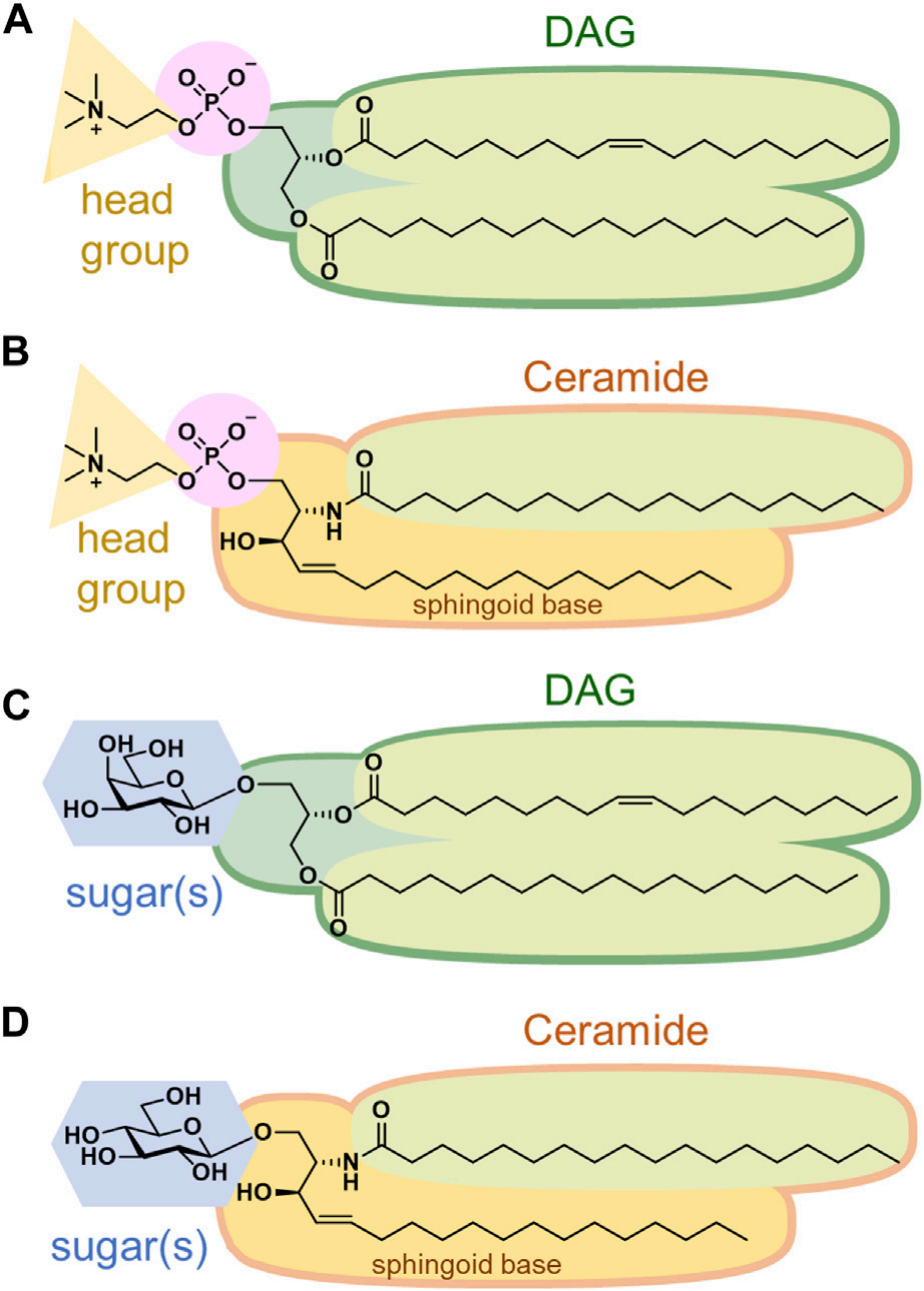
Structures of **(A)** phosphatidylcholine, **(B)** sphingomyelin, **(C)** monogalactosyldiacylglycerol, and **(D)** glucosylceramide.

Phospholipids, the most abundant, are further categorized into glycerophospholipids and sphingophospholipids (Casares et al., 2019). Glycerophospholipids are composed of phosphatidic acid (PA), in which fatty acids are esterified to the C1 and C2 positions of glycerol and a phosphate group is esterified to the C3 position. Saturated fatty acids are often attached at the C1 position, whereas unsaturated fatty acids are attached at the C2 position. The chain length of fatty acids generally falls within the range of 16–20 carbon atoms; however, some cells and microorganisms may contain fatty acids with longer chains. In archaeal cell membranes, ether-type lipids exist, where isoprenoid hydrocarbon alcohols, such as archaeol and cardarchaeol, are linked via an ether bond. Remarkably, the stereochemistry of glycerol in archaea is reversed; a phosphate head group is present at the C1 position. In biological membranes, most phospholipids have polar head groups and only trace amounts are present as PA. Phosphatidylcholine (PC) is the major component of eukaryotic membranes (Figure 2A), followed by phosphatidylethanolamine (PE), phosphatidylglycerol (PG), phosphatidylserine (PS), and phosphatidylinositol (PI). Prokaryotic membranes do not contain PC; however, PE and PG are major components. Cardiolipin (CL), in which two PA moieties connect with a glycerol backbone in the center to form a dimeric structure, is found in the inner mitochondrial membrane and in bacteria.

Sphingoid base, which forms the backbone of sphingolipids, is a long-chain aliphatic amine containing 2–3 hydroxy groups. Sphingosine is the most abundant sphingoid base in mammalian cells. Ceramides are formed when fatty acids attach to the amino groups of sphingoid bases. Sphingomyelin, an emblematic sphingolipid, contains phosphocholine in the primary hydroxy group of ceramide (Figure 2B). Sphingomyelin accounts for 2%–15% of the total phospholipids in mammalian cells (Casares et al., 2019). In addition, sphingolipids with phosphoethanolamine or phosphonoethanolamine (C-P bond) esterified to ceramide are found in lower animals. Yeasts, molds, and plants do not contain sphingomyelin but do contain phosphoinositol-containing sphingolipids, which are not found in higher animals (Sperling and Heinz, 2003). Bacteria, with a few exceptions, do not contain any sphingolipids.

Membrane glycolipids are also classified into glyceroglycolipids and sphingoglycolipids. Glyceroglycolipids are composed of 1–4 sugars linked to the primary hydroxy group of diacylglycerol (DAG). Galactose and fucose are the most common sugars, some of which are sulfated. They are widely distributed in plants, microorganisms, and some animal organs, such as the testes. Monogalactosyldiacylglycerol (MGDG) and digalactosyldiacylglycerol (DGDG) are the main components of the thylakoid membranes, which are the sites of photosynthesis in cyanobacteria and plants (Figure 2C). These two galactolipids account for approximately 80% of the lipids in the thylakoid membranes. In addition, sulfoquinovosyldiacylglycerol (SQDG), a glycolipid containing sulfate esters, accounts for approximately 10%, while phospholipids only account for the remaining 10%.

Sphingoglycolipids are sugar-bound ceramides (Ishibashi et al., 2013). Several sugars, including galactose, glucose, and sialic acid, are present in various arrangements (Figure 2D). Glycan structures are diverse and range from monosaccharides to dozens of sugars. The groups containing sialic acid are referred to as gangliosides. Some sphingolipids undergo sulfation, and more than 400 types of sphingolipids have been identified to date.

In addition, biological membranes contain many trace components (Figure 1, right panel). This review focuses on glycero-glycophospholipids, which have characteristics of both glycerophospholipids and glyceroglycolipids. Glycero-glycophospholipids contain PA as their basic backbone and sugar(s) as the head group. Phosphoglycolipids, which are glycolipids modified with phosphoric acid, have a similar structure, but they belong to a different category. Recently, the unique biological activities of glycero-glycophospholipids, such as involvement in cell differentiation, apoptosis, and bacterial pathogenesis, have attracted attention; however, their scarcity has hampered investigations into their functions. Unlike proteins that can be produced and modified using established molecular biological techniques, the biosynthesis of glycolipids is sometimes difficult because multiple synthetic enzymes are required. Therefore, a chemical synthesis that can supply structurally modified molecules would be useful. Herein, we describe the structure, distribution, function, biosynthesis, and chemical synthesis of representative glycero-glycophospholipids. Moreover, we introduce our recent studies on the rare glycero-glyco“pyrophospho”lipid, which contains a pyrophosphate instead of a phosphate.

## 2 Phosphatidylglucoside (PtdGlc)

### 2.1 Structure, distribution, and functions of PtdGlc

**FIGURE 3:**
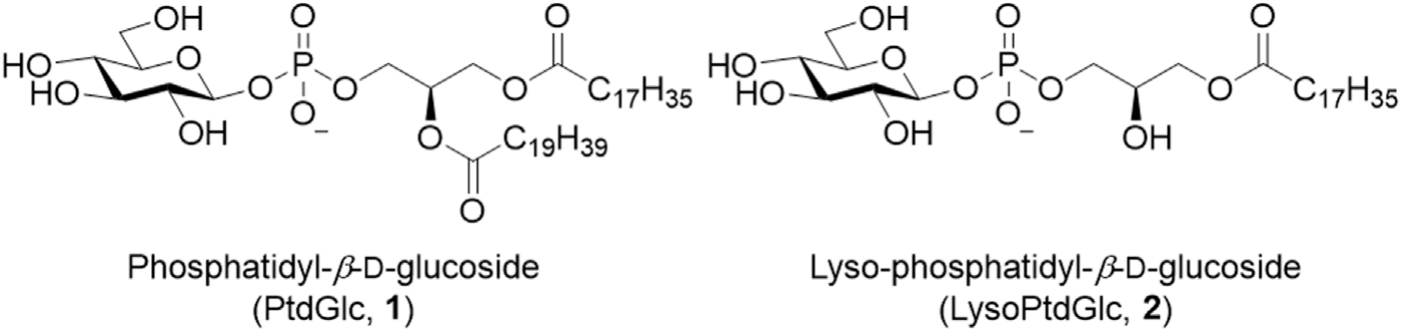
Structures of PtdGlc (**1**) and LysoPtdGlc (**2**).

The simplest glycero-glycophospholipid, phosphatidyl α-glucoside, was identified between 1970 and 1972 from several bacterial species (Shaw et al., 1970; Short and White, 1970; Shaw et al., 1972). In addition, its β-isomer, phosphatidylglucoside (PtdGlc, **1**), was detected in human cord red cells in 2001 (Nagatsuka et al., 2001) (Figure 3). Later, it was purified from fetal mouse brains using a specific monoclonal antibody immunized with detergent-insoluble membranes. The lipid extract from brains was treated with PI-specific phospholipase C to remove PI and was fractionated by using successive column chromatography to give a single spot on thin layer chromatography (TLC). Its structure was unambiguously determined by nuclear magnetic resonance (NMR), gas chromatography (GC), and Fourier transform mass spectrometry (FT-MS) (Nagatsuka et al., 2006; Takahashi et al., 2012). Simultaneously, an analog whose 6-position on glucose was *O*-acetylated was identified. Notably, with respect to the stereochemistry of the glycerol backbone in PtdGlc, the *S* isomer, which is rarely found in mammals, is present in approximately 15% of the PtdGlc. Moreover, the fatty acid composition of PtdGlc is limited to 18:0 at *sn*-1 position and 20:0 at *sn*-2 position, whereas that of ordinary glycerophospholipids is a mixture of several species, depending on the biomembrane environment. It is unusual that both fatty acids are saturated. As might be inferred from its lipid composition, PtdGlc forms microdomains on the plasma membrane, which have been shown to differ in function from the rafts formed by sphingolipids (Murate et al., 2010). The expression of PtdGlc in the central nervous system is cell type- and developmental stage-specific; it is expressed in immature neural stem cells and is involved in their differentiation into astroglia (Kinoshita et al., 2009). PtdGlc is strongly expressed in embryonic rat astroglia. However, its expression is reduced and limited to adult cells. Neurons are thought to hardly synthesize PtdGlc. PtdGlc is also expressed in human neutrophils and is involved in neutrophil differentiation and apoptosis at the final stage (Oka et al., 2009; Kina et al., 2011). Furthermore, PtdGlc can be hydrolyzed in glial membranes and released as LysoPtdGlc (**2**) (Figure 3), a molecular species in which the fatty acid at the *sn*-2 position of glycerol is removed. LysoPtdGlc acts as an intercellular signaling molecule that regulates glia-neuron intercellular communication via the G protein-coupled receptor GPR55 to guide nociceptive axons in the central nervous system (Guy et al., 2015; Shimai et al., 2023).

### 2.2 Biosynthesis of PtdGlc

PtdGlc is synthesized in the endoplasmic reticulum (ER) via a UDP-glucose-dependent reaction. Since glucosylceramide synthase showed no PtdGlc synthesis activity, another β-glucosyltransferase was expected to exist. It was reported that UDP-glucose:glycoprotein glucosyltransferase 2 (UGGT2) produces PtdGlc by using a PA with a saturated fatty acid acyl chain (sPA) as a physiological substrate (Hung et al., 2022). UGGT2 exists in ER membranes as well as in the ER lumen. Remarkably, UGGT2 does not accept unsaturated fatty acid-containing PAs as substrates. Saturated fatty acids (e.g., C18:0-CoA) are converted into unsaturated fatty acids (e.g., C18:1-CoA) by an oxygen-dependent enzyme, namely, ER-associated stearoyl-CoA desaturase (SCD). Suppression of SCD under hypoxic conditions results in an increase in sPA with cytotoxic activity. UGGT2 synthesizes PtdGlc by using sPA, which is concentrated in lysosomes and degraded by the autophagy pathway, thereby avoiding ER stress.

### 2.3 Chemical synthesis of PtdGlc

**SCHEME 1:**
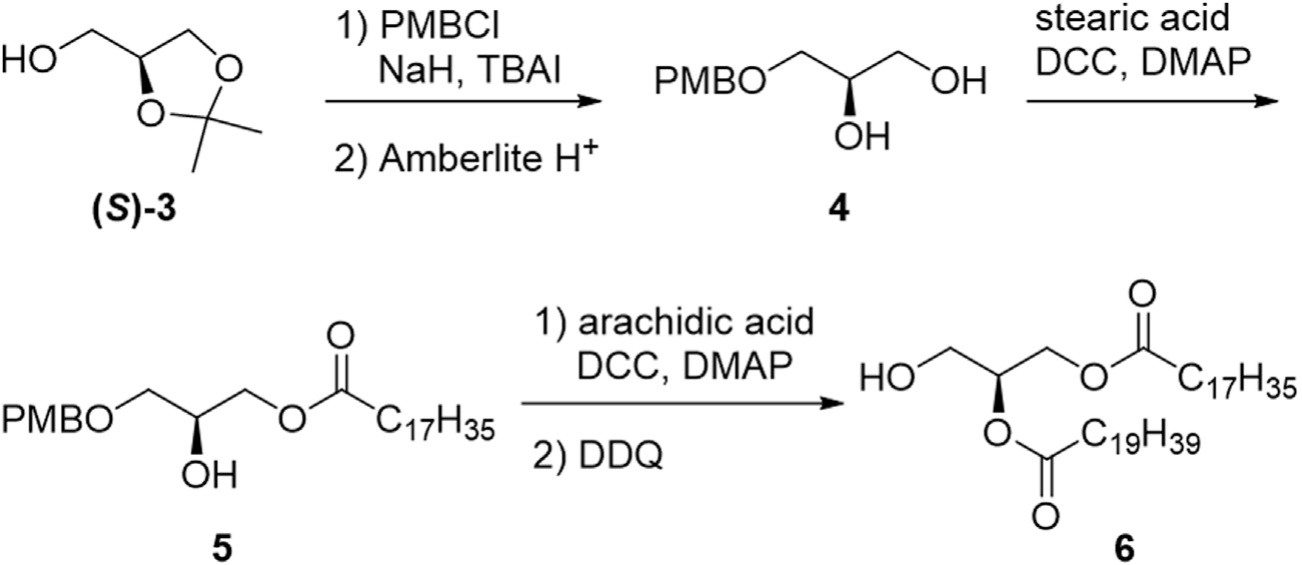
Synthesis of diacylglycerol moiety (**6**).

In the synthetic approach for phosphatidyl-1-D-glucose, an intermediate protected by benzyl groups was synthesized; however, the configuration of the 1-position of glucose was not determined, and deprotection of the benzyl groups was not successful (Ramirez et al., 1983). The first stereoselective chemical synthesis of PtdGlc (**1**) was achieved in 2008 (Greimel and Ito, 2008). Each enantiomer of solketal [(*S*)-**3** or (*R*)-**3**] was used as the starting material to yield chiral DAG [(*S*)-**6** or (*R*)-**6**], respectively (Scheme 1).

**SCHEME 2:**
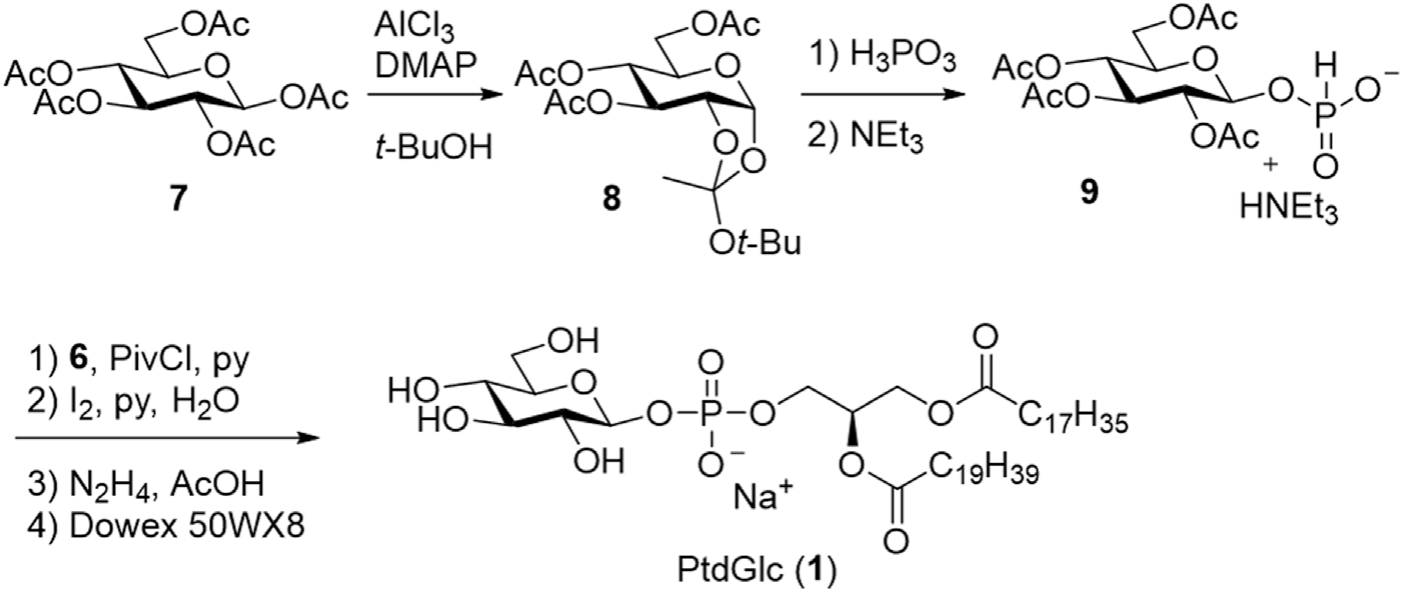
First chemical synthesis of PtdGlc (**1**).

Based on the results obtained by Ramirez et al., the acetyl (Ac) group was selected as the protecting group for the sugar moiety (Scheme 2). Per-acetylated glucose (**7**) was selectively converted into β-phosphonic acid (**9**) via *t*-butyl orthoester (**8**) using phosphonic acid as an autocatalyst. Subsequently, the DAG moiety (**6**) was introduced, and the phosphonate was oxidized to the phosphate diester. Finally, Ac groups were deprotected to give the desired PtdGlc (**1**). Notably, stereoselective synthesis allowed the utilization of both stereoisomers as standards, which verified the presence of both diastereomers in naturally occurring PtdGlc isolated from mammalian cells.

**SCHEME 3:**
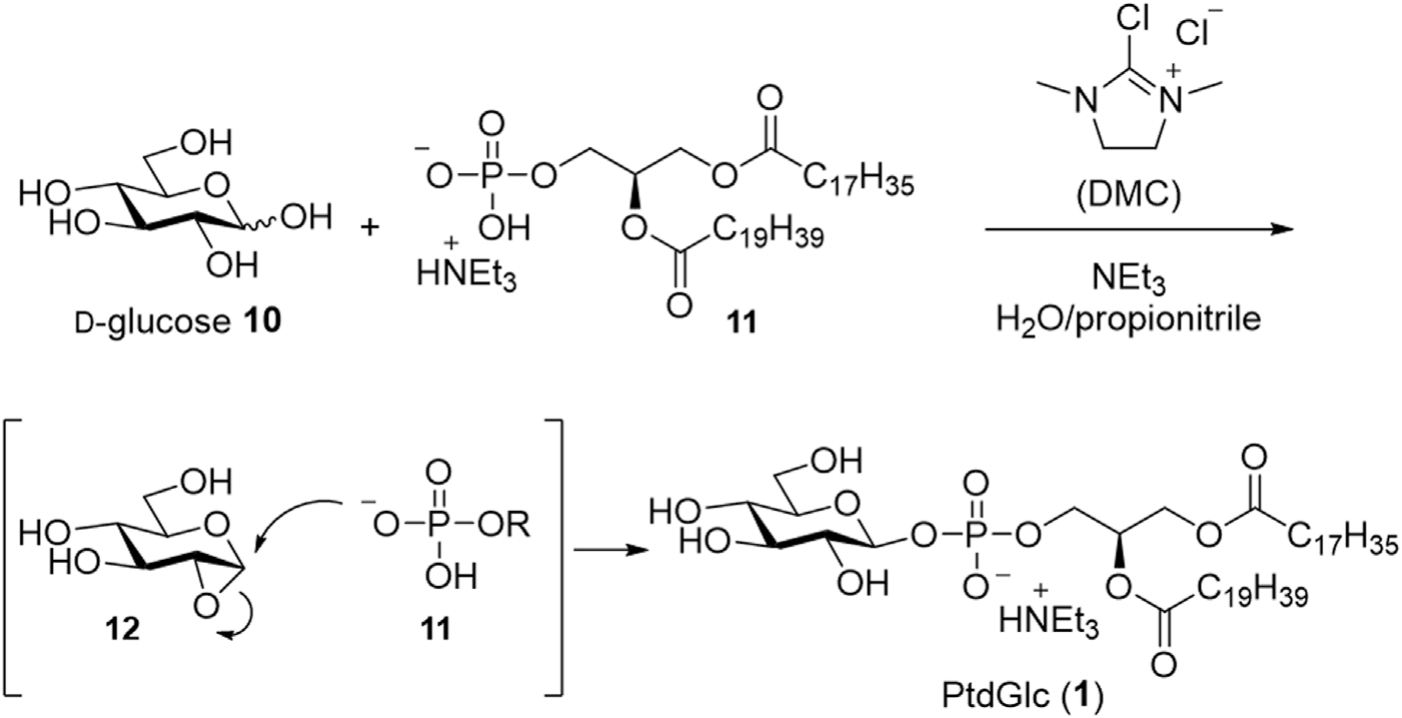
Protection-free synthesis in aqueous media.

Recently, protection-free synthesis of PtdGlc in aqueous media was reported (Kano et al., 2023a; Kano et al., 2023b) (Scheme 3). The β-glucosyl phosphate linkage was successfully constructed from phospholipid and D-glucose using 2-chloro-1,3-dimethylimidazolinium chloride (DMC) as a condensing reagent and triethylamine in a mixture of water and propionitrile. Subsequently, PtdGlc (**1**) was successfully obtained via only two purification steps: silica gel column chromatography and gel permeation column chromatography.

PtdGlc can be enzymatically synthesized from D-glucose and PC (Inoue et al., 2016). In addition, several analogs have been chemically synthesized; the glucose moiety of PtdGlc has been converted into other monosaccharide components, or the phosphate group has been converted into thiophosphate (Greimel et al., 2008). The synthesis of LysoPtdGlc (**2**) (Kano et al., 2021) and its analog, in which the phosphate moiety is converted into a squaryl diamide group, has also been reported (Ding et al., 2018). The biological activities of PtdGlc (**1**) and LysoPtdGlc (**2**) have been investigated using these analogs.

## 3 Enterobacterial common antigen (ECA) and membrane protein integrase (MPIase)

### 3.1 Structure, distribution, and functions of ECA

**FIGURE 4:**
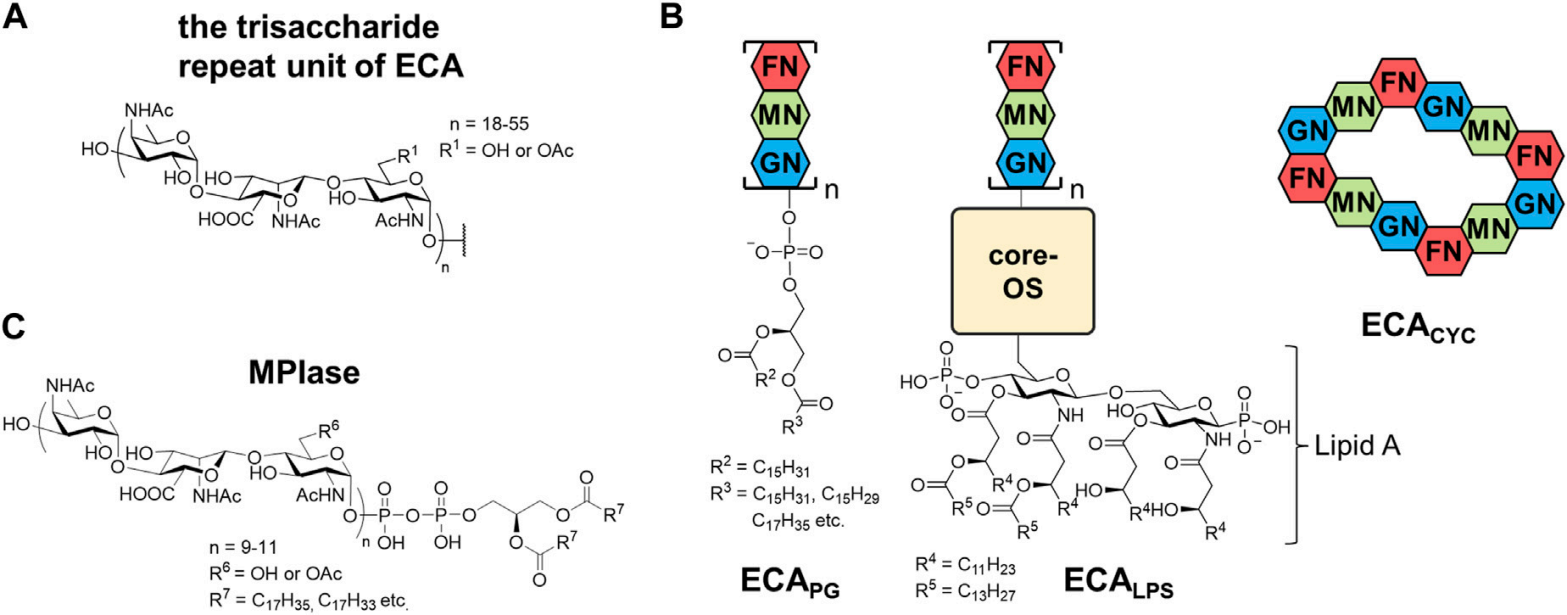
Structures of **(A)** a trisaccharide unit of ECA, **(B)** three types of ECA, and **(C)** MPIase. GN, *N*-acetylglucosamine; MN, *N*-acetyl-D-mannosaminuronic acid; FN, 4-acetamido-4,6-dideoxy-D-galactose; OS, oligosaccharides.

Enterobacterial Common Antigen (ECA) is a glycero-glycophospholipid possessing a long sugar chain and is found on the outer membrane of various enterobacteria, including *Escherichia coli, Shigella sonnei,* and *Salmonella enterica* (Mäkelä and Mayer, 1976; Basu et al., 1987; Kuhn et al., 1988; Rai and Mitchell, 2020). ECA was firstly discovered as Kunin antigen which was commonly present in most enterobacteria (Kunin, 1963). ECA polysaccharide chains are composed of the trisaccharide repeat unit; →3)-D-α-Fuc4NAc-(1→4)-β-D-ManNAcA-(1→4)-α-D-GlcNAc(1→, where Fuc4NAc, ManNAcA, GlcNAc are 4-acetamido-4-deoxyfucose, 2-acetamido-2-deoxymannuronic acid, and 2-acetamido-2-deoxyglucose (*N*-acetyl-glucosamine), respectively (Lugowski et al., 1983; Basu et al., 1987; Bruix et al., 1995). The amino group of each sugar is *N*-acetylated, and the 6-position of GlcNAc is partially *O*-acylated (Figure 4A). Initially, only GlcNAc and ManNAcA were characterized by gas-liquid chromatography (GLC)-MS of the methylated components after hydrolysis. However, afterward, very acid-labile Fuc4NAc was detected as an additional component. The anchor moiety was identified as a PA with an α-glycosyl linkage by GLC-MS and sensitivity to phospholipase D (Kuhn et al., 1983; Kuhn et al., 1988). In addition to ECA_PG_, a PA-bound ECA form, there are two different forms. ECA_LPS_, which is attached to the nonreducing terminal sugar of lipid A instead of the *O*-antigen of LPS, is found in the outer membrane (Gozdziewicz et al., 2014; Maciejewska et al., 2020), whereas ECA_CYC_, a cyclized oligosaccharide composed of 4–6 trisaccharide units, is found in the periplasm (Figure 4B). The number of repeats of the ECA_PG_ trisaccharide units was estimated to range from 18 to 55, and the molecular weight distribution was broad. Some studies have reported 1–14 trisaccharide repeats (Barr et al., 1999; Rai and Mitchell, 2020); however, SDS-PAGE showed a ladder with molecular weights above approximately 17 kDa (Barr et al., 1988). Although it varies among bacterial species (Kuhn et al., 1988), there seem to be at least 20 repeats in all species.

Because ECA is not essential for enterobacteria, its function is yet to be clearly understood. Although the physiological roles of ECA may vary across bacterial species, ECA contributes to the resistance to toxic molecules (Barua et al., 2002; Ramos-Morales et al., 2003) and the barrier function of outer membrane (Mitchell et al., 2018; Jiang et al., 2020). Additionally, it has been suggested that ECA is involved in the pathogenicity of enterobacteria. In *Salmonella enterica,* ECA-lacking mutant strain was sensitive to bile salts, powerful disruptors of biological membranes, leading to reduced virulence (Ramos-Morales et al., 2003). Despite infection with *S*. *enterica* serovar Typhimurium causing debilitating inflammatory diarrhea, ECA-lacking mutant strains displayed attenuation *in vivo*. Remarkably, the ECA-deficient strains established a persistent low-level infection and provided protection against a subsequent lethal challenge with wild-type *S.* Typhimurium. (Gilbreath et al., 2012). These findings suggest the possibility of using ECA-negative strains as live attenuated vaccine candidates (Liu et al., 2020).

The immunogenicity differs between ECA forms (Rai and Mitchell, 2020). ECA_PG_ is antigenic but not immunogenic; the production of antibodies requires a concomitant adjuvant or protein antigen. In contrast, ECA_LPS_ stimulates the antibody production because it possesses an intrinsic adjuvant Lipid A in its structure (Maciejewska et al., 2020). ECA antibodies have been detected in serum following infections by pathogenic bacteria, and thus, they are useful for serodiagnosis.

### 3.2 Structure, distribution, and functions of MPIase

Membrane protein integrase (MPIase) is a glycolipid found in the inner membrane of *E. coli* (Figure 4C) (Fujikawa et al., 2022; Nishikawa et al., 2022). Although its name is unlikely to indicate a glycolipid, it is derived from the unique activity involved in membrane protein integration (Nishiyama et al., 2006; Nishiyama et al., 2010; Nishiyama et al., 2012). During membrane protein integration, the nascent protein generated from a ribosome is targeted to the membrane, and the first transmembrane region is inserted into the membrane via protein complex channels called the Sec translocon (Sec-dependent pathway). MPIase works with Sec translocons and/or the membrane chaperone YidC to promote Sec-dependent integration of membrane proteins and translocation of secretory proteins (Nishiyama et al., 2006). Additionally, a Sec-independent pathway is known for a subset of small membrane proteins. MPIase is required for Sec-independent integration into the *E. coli* inner membrane (Nishiyama et al., 2006). Although the Sec translocon is conserved in all organisms, the presence of MPIase in species other than *E. coli* has not yet been investigated.

MPIase was discovered in the process of studying the *in vitro* reconstitution system of Sec translocon. Since an extract of the *E. coli* inner membrane vesicles that did not contain proteinaceous integration factors showed the membrane protein integration activity in *in vitro* translation system, it was fractionated through several column chromatography including a liquid-liquid partition chromatography. NMR measurements and amino acid analysis of purified MPIase indicated that it contains only sugars and lipids. Matrix assisted laser desorption/ionization (MALDI) MS analyses suggested that MPIase is a mixture of homologs with diversity in the length of glycan, acetyl group modification, and fatty acid components. Combination of extensive instrumental analyses, such as MS/MS, GC-MS, and two-dimensional NMR, and the comparison with synthetic substructures revealed that the glycan moiety of MPIase is the same as that of ECA; it consists of repeating trisaccharide units →3)-D-α-Fuc4NAc-(1→4)-β-D-ManNAcA-(1→4)-α-D-GlcNAc-(1→. However, ^31^P-NMR indicated that MPIase is connected to DAG by a pyrophosphate instead of a phosphate (Nishiyama et al., 2012). Glycolipids with pyrophosphorylated DAG are extremely rare, and those found to date are likely the biosynthetic intermediates of MPIase (Rick et al., 1998; Jiang et al., 2020). Similar to ECA, approximately one-third of the 6-hydroxy group in GlcNAc is randomly *O*-acetylated. The number of repeats in a trisaccharide unit is 9–11, which are much fewer and less variable than that of ECA. The fatty acid composition of DAG is similar to that of other phospholipids in the inner membrane of *E. coli* and varies depending on the growth environment.

### 3.3 Biosynthesis of ECA

**FIGURE 5:**
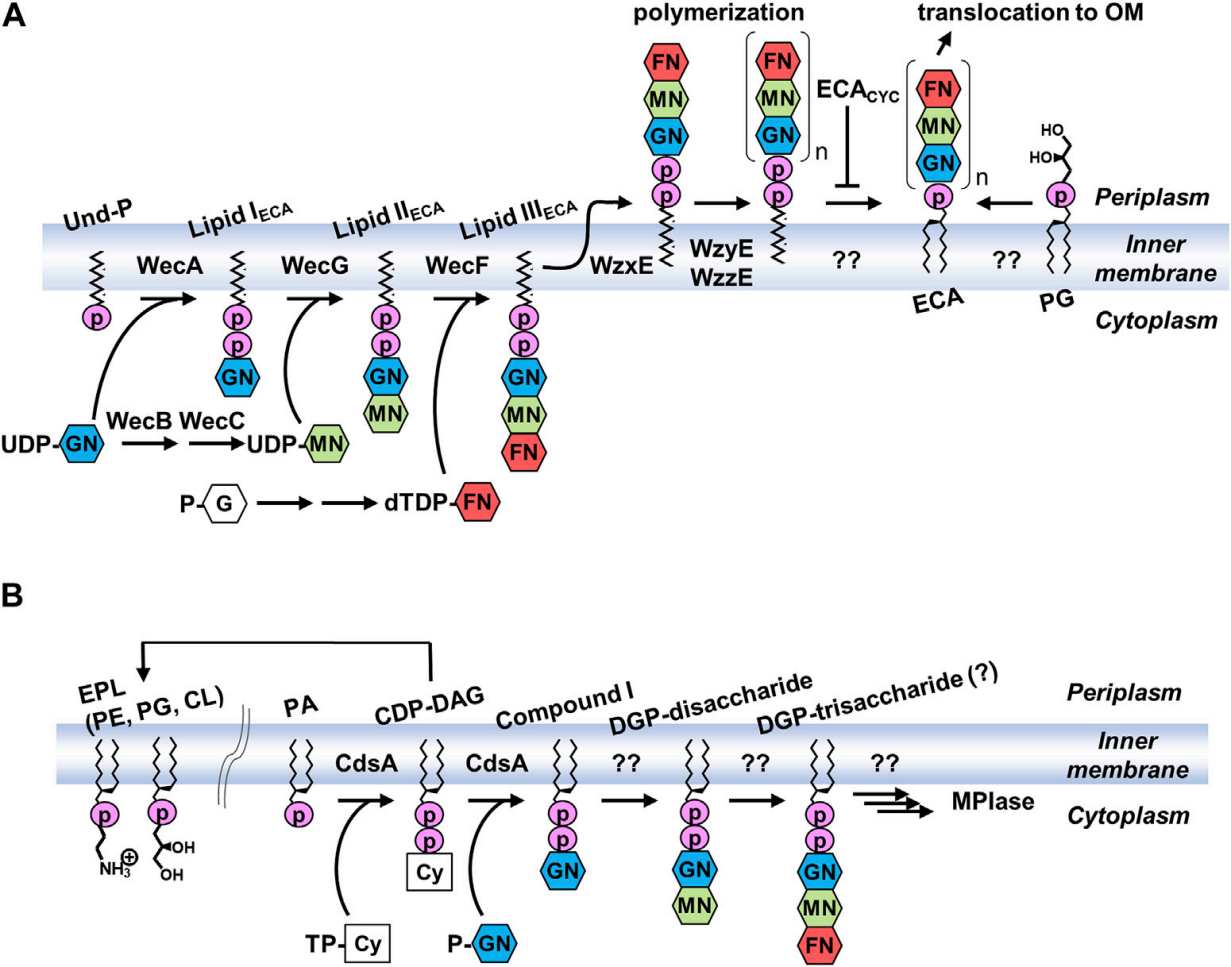
Biosynthetic pathways of **(A)** ECA and **(B)** MPIase. PG, glucose 1-phosphate; UDP, uridine diphosphate; dTDP, thymidine diphosphate; TP-Cy, Cytidine triphosphate; P-GN, *N*-acetylglucosamine 1-phosphate; G, glucose; GN, *N*-acetylglucosamine; MN, *N*-acetyl-D-mannosaminuronic acid; FN, 4-acetamido-4,6-dideoxy-D-galactose; CDP-DAG, cytidine diphosphate diacylglycerol; DGP, diacylglycerol pyrophosphate.

Genes required for multiple steps in ECA biosynthesis are located within the *wec* operon (Meier-Dieter et al., 1990; Daniels et al., 1992; Meier-Dieter et al., 1992) (Figure 5A). The ECA trisaccharide repeating unit is constructed on a lipid carrier, undecaprenyl phosphate (Und-P) (Rick et al., 1998). Und-P consists of phosphate and undecaprenol (Und), a 55-carbon molecule composed of isoprenoid units. Und-P is a universal lipid carrier required for the synthesis of glycans, and, in addition to ECA, it is used for the production of *O*-antigens, peptidoglycans, and capsules in *E. coli* (Rick et al., 1977). The first step is the synthesis of the lipid I_ECA_ using UDP-GlcNAc and Und-P as substrates, which is catalyzed by WecA (Barr and Rick, 1987). Subsequently, WecG adds UDP-ManNAcA to the lipid I_ECA_ to produce the lipid II_ECA_ (Barr and Rick, 1987; Barr et al., 1988; Barr et al., 1989). UDP-ManNAcA, which is rarely found in eukaryotes, is synthesized from UDP-GlcNAc using WecB and WecC (Meier-Dieter et al., 1990). The third sugar is added by WecF using dTDP-FucNAc as a substrate to form lipid III_ECA_, a trisaccharide linked to Und via pyrophosphate (Barr et al., 1989). Lipid III_ECA_ is synthesized in the inner leaflet of the inner membrane facing the cytoplasm. In contrast, the polymerization of trisaccharide units occurs on the periplasmic side of the inner membrane. Thus, lipid III_ECA_ is reversed by the flippase WzxE (Rick et al., 2003). The trisaccharide units are then polymerized by WzyE on the Und-PP carrier to form long sugar chains, the chain length of which is determined by the chain length modulation factor, WzzE (Barr et al., 1999; Kajimura et al., 2005). The hydroxy group at the 6-position of the GlcNAc residues in both ECA_CYC_ and ECA_PG_ is non-stoichiometrically *O*-acetylated by WecH (Kajimura et al., 2006). Recently, it was proposed that ECA_CYC_, together with an inner membrane protein ElyC, acts to regulate the reaction that removes polymerized glycan from Und-PP and forms ECA_PG_ (Rai et al., 2021). The level of ECA_CYC_ in periplasm can be assessed by ElyC to provide feedback regulation. The lipid donor was identified as PG, but the enzyme that catalyzes this reaction is yet to be identified (Morris and Mitchell, 2023). Biosynthesis after the polymerization of trisaccharide units also occurs in the outer leaflet of the inner membrane, but the final product exists in the outer leaflet of the outer membrane. However, the mechanisms underlying this translocation remain unclear. WaaL is involved in the biosynthesis of ECA_LPS_ (Pinta et al., 2012), whereas WzzE is involved in ECA_CYC_ biosynthesis (Kajimura et al., 2005), and deletion of these genes resulted in the loss of these forms of ECA.

Because the biosynthesis of other glycans, such as peptidoglycan and *O*-antigen, also uses Und-P as a carrier, loss of the common pool of Und-P causes cell abnormalities, such as filamentation and swelling. When the biosynthesis of dTDP-Fuc4NAc was interrupted, the accumulation of lipid II_ECA_ lead to a decrease in free Und-P and bacterial cell shape alteration due to restricted peptidoglycan synthesis (Jorgenson et al., 2016). The lack of the flippase WzxE or trisaccharide polymerase WzyE resulted in the accumulation of lipid III_ECA_, which is lethal to cells (Rick et al., 2003; Kajimura et al., 2005).

### 3.4 Biosynthesis of MPIase

The trisaccharide moiety of MPIase is the same as that of ECA; however, surprisingly, MPIase is produced even in ECA biosynthetic enzyme-lacking *E. coli* (Kamemoto et al., 2020). Conversely, ECA was normally expressed upon MPIase depletion. Therefore, a biosynthetic pathway different from that of ECA is presumed to exist (Figure 5B). When Fuc4NAc was depleted during ECA biosynthesis, 1,2-diacyl-*sn*-glycero-3-pyrophosphoryl-GlcNAc-ManNAcA (DGP-disaccharide), in which the disaccharide is attached to DAG via pyrophosphate, was found to be associated with Lipid II_ECA_ (Rick et al., 1998). This may be a biosynthetic intermediate for MPIase, suggesting that there is a system that directly uses DAG rather than Und as an anchor to elongate the glycan chain. There are no other examples in which a DAG was initially used as a carrier. A search for the enzymes responsible revealed that CdsA and its paralog, YnbB, are involved in the production of GlcNAc-PP-DAG (compound I) (Sawasato et al., 2019a; Sato et al., 2019). CdsA is known as the enzyme that synthesizes cytidine diphosphate diacylglycerol (CDP-DAG), an important biosynthetic intermediate of membrane lipids from PA (Icho et al., 1985). When the inner membrane vesicles prepared from CdsA-overexpressing cells were incubated with [^14^C]PA, CTP, and possible GlcNAc donors [UDP-GlcNAc, CDP-GlcNAc, and GlcNAc-1-phosphate (GlcNAc-P)], compound I was produced in a GlcNAc-P-dependent manner (Sawasato et al., 2019a). When CdsA was deficient in *E. coli*, MPIase depletion, abnormal protein membrane transport, and inhibition of bacterial growth were observed, even when membrane lipids syntheses were compensated for by the mitochondrial CDP-DAG synthase Tam41P (Tamura et al., 2013). CdsA and YnbB are temperature-sensitive, and incubation at low temperatures (<25°C) elicited an upregulation of these genes and increased production of MPIase (Sawasato et al., 2019b). The biosynthetic enzymes responsible for the condensation of the second sugar and the subsequent reactions are not known. Based on its function, MPIase is thought to be present in the inner leaflet of the inner membrane; however, its distribution in bacteria remains unknown. Identification of biosynthetic enzymes is essential for understanding the localization and function of MPIase *in vivo*.

### 3.5 Chemical syntheses of MPIase analogs

**FIGURE 6:**
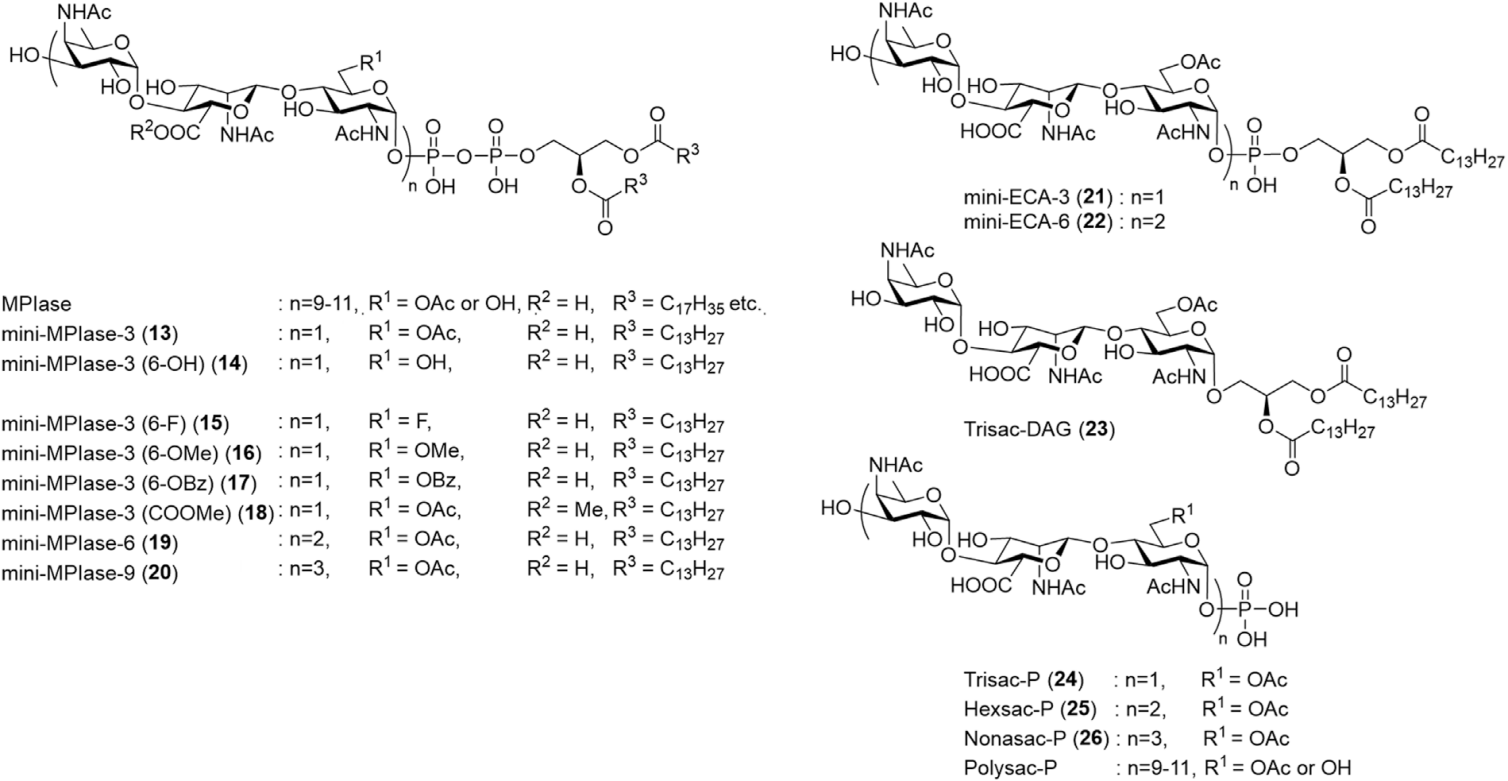
Chemically synthesized MPIase analogs.

Chemical syntheses of the trisaccharide and hexasaccharide moieties of ECA, which have the same glycan structure as MPIase, were reported (Paulsen and Lorentzen, 1985; Liu et al., 2015); however, these did not contain the phospholipid moiety. The first synthesis of MPIase analog containing a pyrophospholipid moiety was achieved using a minimal structure called mini-MPIase-3 (**13**) (Fujikawa et al., 2018). Mini-MPIase-3 exhibited membrane protein integration activity, indicating that glycolipids underlie membrane protein integration. Following that, various analogs shown in Figure 6 were synthesized to elucidate the action mechanism of MPIase: glycan length variants [mini-MPIase-9 (**20**), mini-MPIase-6 (**19**), mini-MPIase-3 (**13**), Nonasac-P (**26**), Hexsac-P (**25**), Trisac-P (**24**)], modification of the functional group at C6 of GlcNAc [mini-MPIase-3 (6-OH) (**14**), mini-MPIase-3 (6-F) (**15**), mini-MPIase-3 (6-OMe) (**16**), mini-MPIase-3 (6-OBz) (**17**)] or the carboxy group of ManNAcA [mini-MPIase-3 (COOMe) (**18**)], and variants for the pyrophosphate moiety [mini-ECA-6 (**22**), mini-ECA-3 (**21**), Trisac-DAG (**23**)] (Fujikawa et al., 2023).

**SCHEME 4:**
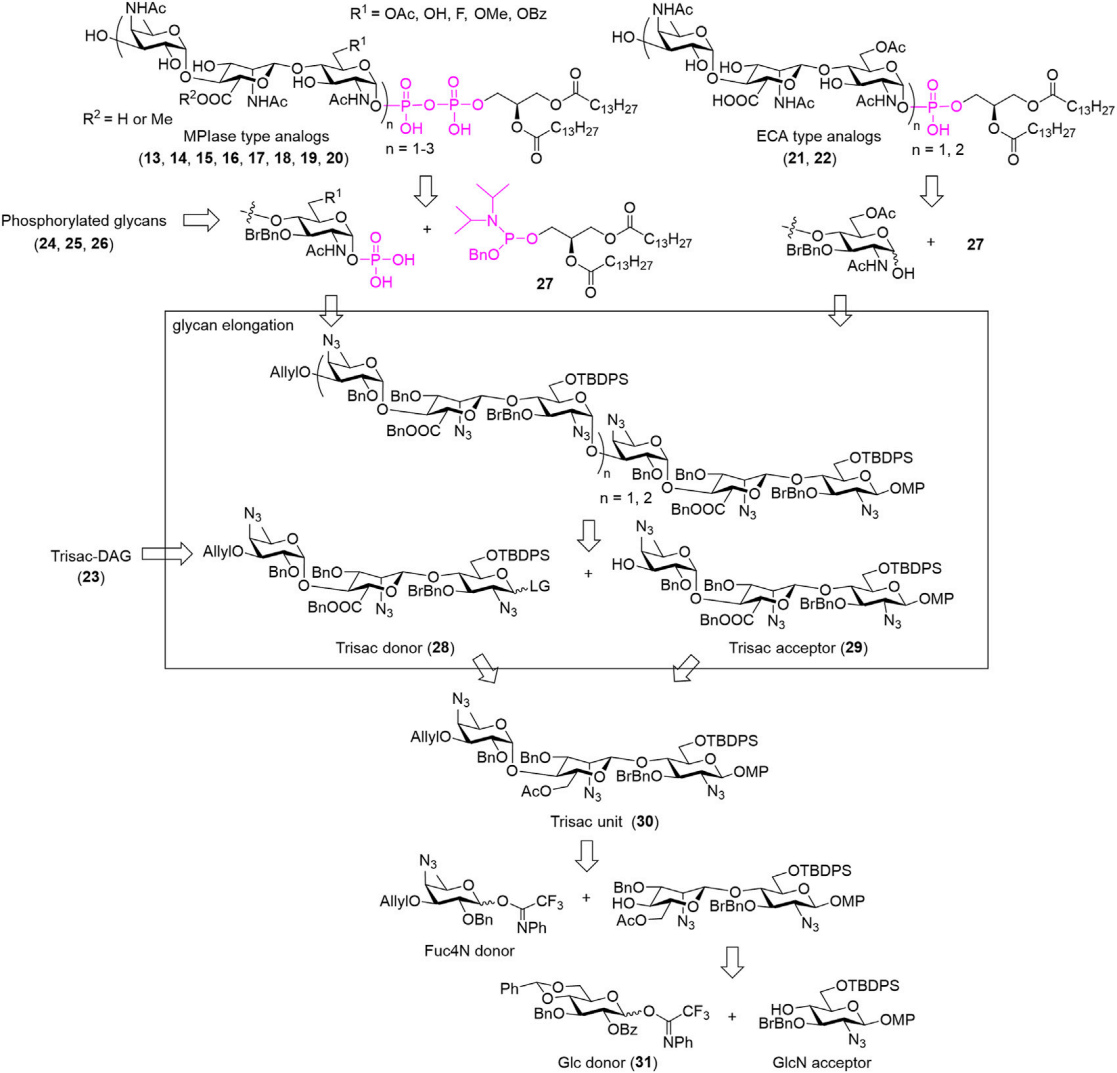
Retrosynthetic strategy for MPIase analogs.

A retrosynthetic strategy for the MPIase analogs is shown in Scheme 4. An unstable phospholipid moiety is introduced at the end of the synthesis process. The MPIase-type (**13–20**) with a pyrophosphate is constructed by the condensation of the lipidated phosphoramidite reagent (**27**) and the monophosphoryl glycan, whereas the ECA-type (**21**, **22**) with a monophospholipid is constructed by introducing **27** into the hemiacetal form of the glycan. Because MPIase has a repeating trisaccharide structure, glycan elongation is performed based on the trisaccharide unit. The common trisaccharide intermediate (**30**) for both the donor (**28**) and acceptor (**29**) is designed as follows: For the construction of the α-glucosaminide bond between the trisaccharide units, the GlcNAc donor has an azide group without a neighboring participation effect at C2, bulky *p*-BrBn at O3, and TBDPS groups at O6 (Zulueta et al., 2012; Zulueta et al., 2013). The TBDPS group is also useful for selective deprotection because the O6 of GlcNAc is converted into the Ac group or other substituents at a late stage of the synthesis. Orthogonal deprotection of an allyl group at the nonreducing end or a methoxyphenyl (MP) group at the reducing end enables the conversion of **30** to the donor (**28**) or acceptor (**29**), respectively. The β-mannosaminide bond in the trisaccharide unit is constructed by steric inversion of C2 of Glc with NaN_3_ after obtaining the β-glucoside using neighboring-group participation of the 2-OBz group in the Glc donor (**31**) (Nishiyama et al., 2012).

### 3.6 Mechanism of membrane protein integration of MPIase

**FIGURE 7:**
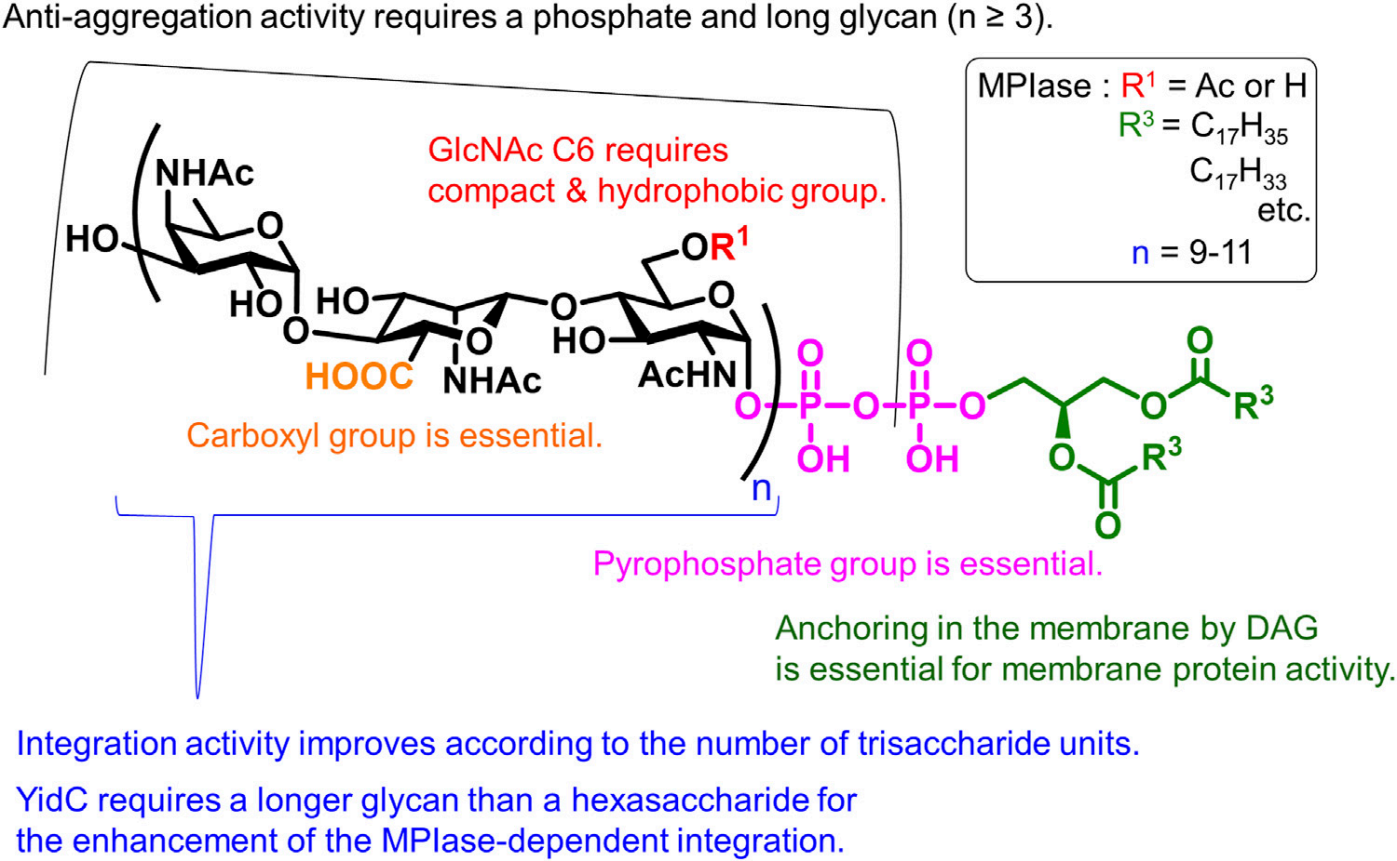
Structural requirements of MPIase.

The synthetic MPIase analogs enabled elucidation of the mechanism of the Sec-independent pathway. The membrane protein integration activity of the analog was evaluated using liposome membranes and model proteins produced using an *in vitro* translation system (Koch et al., 1999; Nishiyama et al., 2006; Nishiyama et al., 2010). Liposomes were prepared from *E. coli* phospholipids (PE, PG, and CL) and DAG, in which spontaneous protein integration was completely inhibited (Kawashima et al., 2008). When natural MPIase was incorporated into liposomes, Sec-independent membrane protein integration was observed in a dose-dependent manner (Nishiyama et al., 2006; Kawashima et al., 2008; Nishiyama et al., 2010; Nishiyama et al., 2012; Nishikawa et al., 2017; Sasaki et al., 2019; Endo et al., 2021); and when SecYEG was incorporated into liposomes together with MPIase, Sec-dependent membrane protein integration was also reproduced (Nishiyama et al., 2006; Sawasato et al., 2019a). When the aforementioned synthetic MPIase analogs were reconstituted into liposomes, membrane protein integration activity was observed according to glycan length. The importance of pyrophosphate was clearly demonstrated, because the activity of mini-ECA-6 (**22**) was weaker than that of mini-MPIase-6 (**19**), reflecting the absence of activity in natural ECA. The glycan moiety of MPIase devoid of the lipid moiety did not exhibit integration activity; however, it showed chaperone-like activity that prevented the aggregation of substrate proteins. Detailed structure–activity relationship studies are summarized in Figure 7 (Fujikawa et al., 2023).

The contributions of the 6-OAc group in GlcNAc, carboxy group in ManNAcA, and pyrophosphate group are indicated. Anchoring in the membrane by a lipid moiety is essential for membrane protein integration. Long glycans enhance activity, and the synergistic effect between MPIase and the membrane chaperone YidC requires longer glycans than hexasaccharides. Furthermore, physicochemical measurements, such as surface plasmon resonance (SPR) and saturation transfer difference- (STD)-NMR, using MPIase analogs have verified the direct interactions between MPIase and the substrate protein (Mori et al., 2022). Both hydrophobic and electrostatic interactions contribute to this activity. Solid-state NMR and fluorescence measurements revealed that MPIase increases membrane mobility and exposes the deep hydrophobic core of the membrane, facilitating the integration of the hydrophobic transmembrane region of substrate proteins into the membrane (Nomura et al., 2019; Nomura et al., 2022).

**FIGURE 8:**
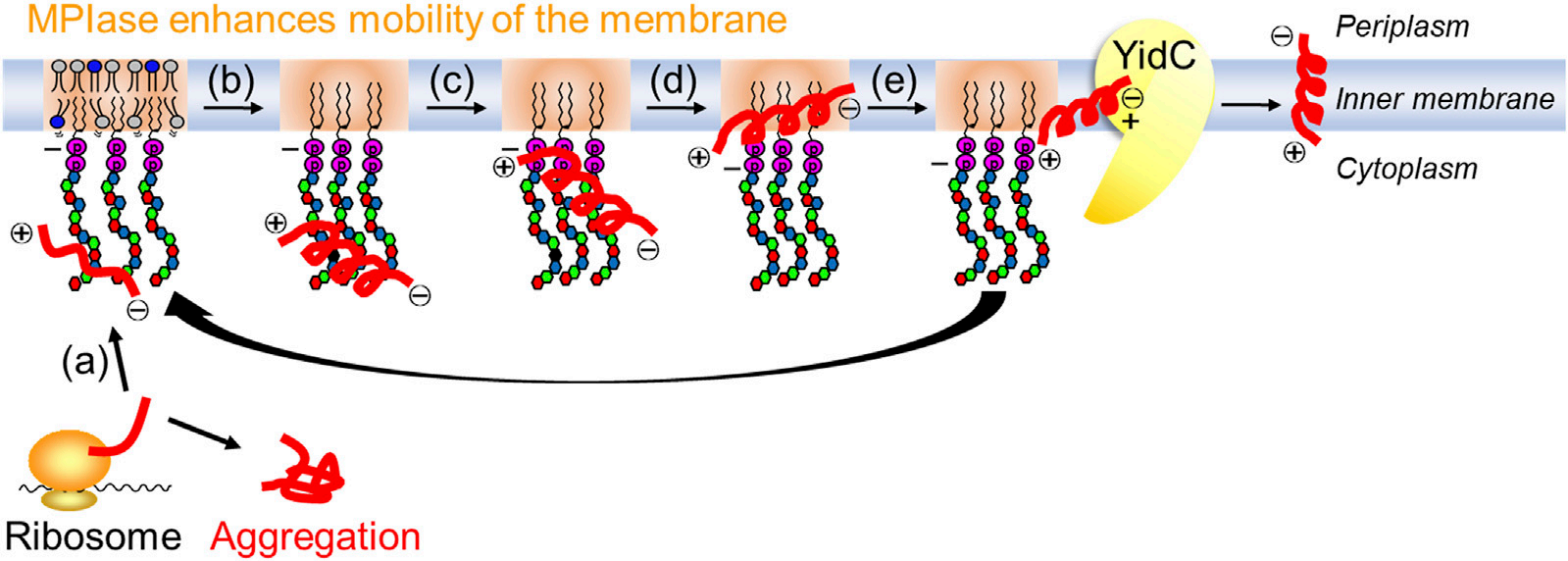
Mechanism for Sec-independent integration involving MPIase. MPIase sequentially (a) captures a substrate protein, (b) inhibits its aggregation, (c) attracts it to the membrane surface, (d) integrates it into the membrane, and (e) transfers it to YidC.

These studies demonstrated the mechanism through which the glycolipid supports the Sec-independent membrane protein integration in the inner membrane of *E. coli*, which is as follows (Figure 8): (a) the glycan moiety of MPIase captures the substrate protein using various functional groups, (b) the secondary structure of the substrate protein is altered to prevent the aggregation of substrate proteins, (c) the strong negative charges of pyrophosphate attract the positive charges of basic amino acid residues, which generally exist in the substrate protein as known “positive inside rule,” to the membrane surface, (d) the hydrophobic interactions between the substrate protein and the membrane induce the substrate protein integration in the membrane core area where the mobility is higher, and (e) MPIase transfers the substrate protein to the membrane chaperone YidC to regenerate the capability of substrate capturing. The localized state of MPIase on the membrane and the cooperation of MPIase with Sec factors are still unresolved and are considered as future issues.

## 4 Lipids with similar structure

The lipids shown here are not glycero-glycophospholipids but have similar structures. They were introduced to compare their structures.

### 4.1 Glycosylphosphatidylinositol anchor

**FIGURE 9:**
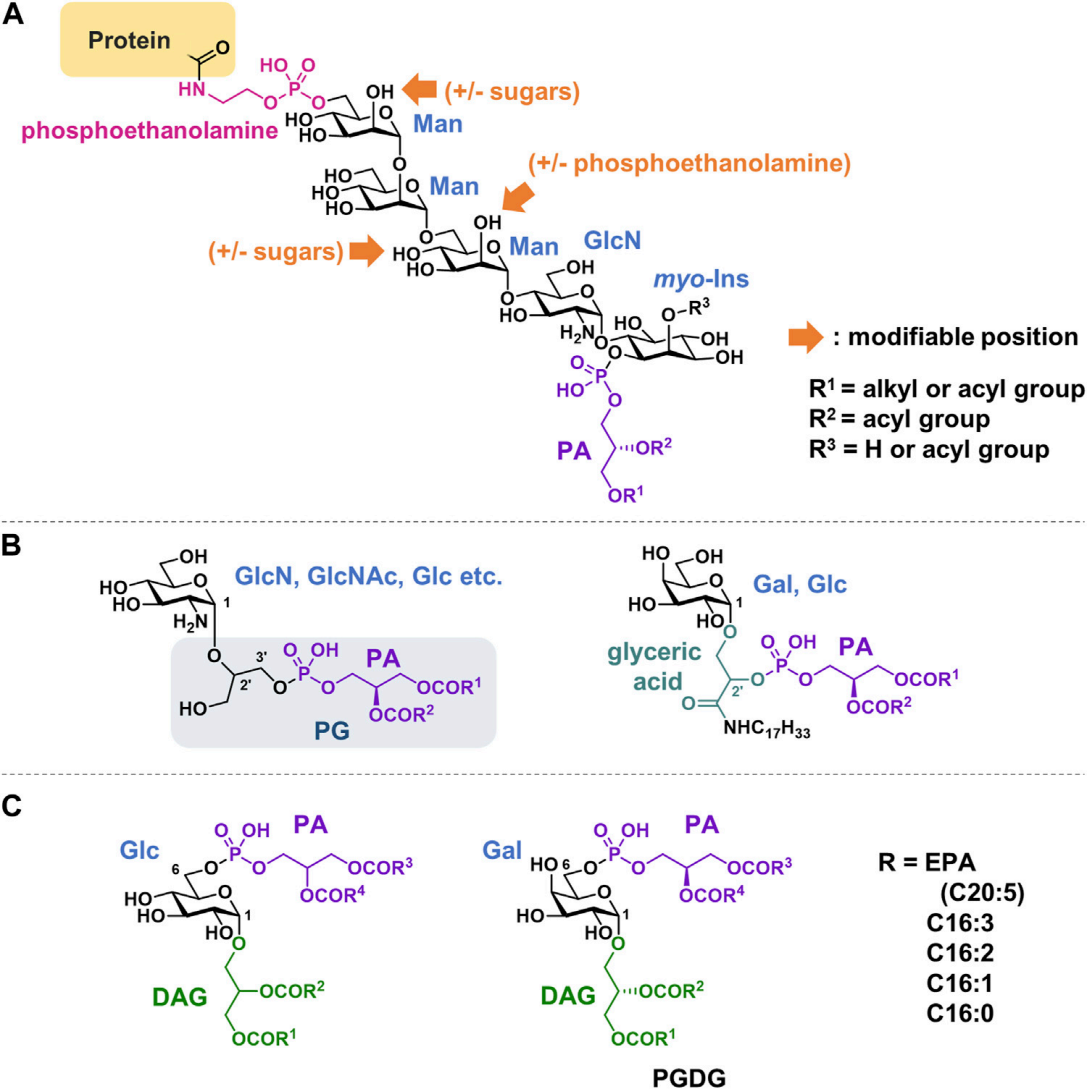
Structures of **(A)** GPI anchor, **(B)** Glycerophospholipids with a linker of glycerol or glyceric acid, **(C)** Glycolipid phosphorylated at 6-position. PA, phosphatidic acid; DAG, diacylglycerol; PG, Phosphatidylglycerol; PGDG, phosphatidylmonogalactosyldiacylglycerol; EPA, eicosapentaenoic acid.

Molecules in which PA is attached to inositol-containing pseudo-glycans are well-known. The most well-known of these is the glycosylphosphatidylinositol (GPI) anchor (Figure 9A). GPI anchors are glycolipids attached to the C-termini of proteins via post-translational modifications. PI is linked to a linear core glycan comprising three mannose (Man) residues and one GlcNAc residue. At the nonreducing end of the glycan, phosphoethanolamine is attached, whose amino group is bound to the C-terminus of the protein. The core glycan can be further modified with a variety of sugars depending on the type of protein. The lipid moiety on mammalian GPI-anchored proteins is mostly of the 1-alkyl-2-acylglycerol type (alkyl type), but some are of the diacylglycerol type (diacyl type). The fatty acid composition is characterized by the presence of saturated fatty acids at the *sn*-2 position of the glycerol backbone.

Each step of GPI-anchor biosynthesis has been studied in detail (Kinoshita, 2020). GPI-modified proteins have a GPI-attaching signal at the C-terminus in addition to an N-terminal ER target signal. The hydrophobic signal sequence is cleaved, and the newly exposed C-terminus is linked to the amino group of the phosphoethanolamine of the anchor via an amide bond.

GPI anchoring is a widely conserved post-translational modification that occurs in eukaryotes. A wide variety of proteins, including receptors, cell adhesion factors, and hydrolytic enzymes, undergo GPI modification and bind to biomembranes. Typically, GPI anchor–protein complexes are associated with raft domains in membranes. Phospholipase C (PLC) cleaves the phosphoglycerol bond of the GPI anchor, causing the release of GPI-binding proteins from the cell membrane.

Chemical synthesis of GPI anchors has been actively studied by many research groups since the 1990s (Murakata and Ogawa, 1991; Mayer et al., 1994; Campbell and Fraser-Reid, 1995; Tailler et al., 1999; Xue and Guo, 2003; Ali et al., 2005), which was summarized in detail in an excelent review (Swarts and Guo, 2012). Synthetic research has not only focused on inherent GPI anchors but has also extended to analogs with labeling probes for clicks (Swarts and Guo, 2011; Ding et al., 2017; Suazo et al., 2021; Yan and Guo, 2023) or photoaffinity reactions (Mullapudi et al., 2022; Kundu et al., 2023).

### 4.2 Glycerophospholipids with a linker of glycerol or glyceric acid

Phosphatidyl glycerol analogs, in which a sugar is attached to glycerol in the head group, have been observed in bacteria (Gurr et al., 1968; Phizackerley and MacDougall, 1968; Peleg and Tietz, 1973) (Figure 9, B). Sugar is thought to enzymatically transfer from UDP-hexose into PG. Examples include glucosamine, galactosamine, GlcNAc, and glucose. Both 2′and 3′positions in the glycerol can be substituted. The substitution of the glycerol moiety in CL was also observed. Phospholipids containing glyceric acids in their head groups have been isolated from bacteria and archaea. Glyceric acid is amidated with long-chain amines (Anderson and Hansen, 1985; Huang and Anderson, 1989). These compounds are suggested to stabilize the membranes of thermophilic bacteria when exposed to high temperatures.

### 4.3 Glycolipids phosphorylated at 6-position

There are some glycolipids in which PA is not glycosylated at the 1-position of the sugar, but rather binds to the hydroxy group at the 6-position (Figure 9C). These compounds can be classified as phosphorylated glyceroglycolipids. Glucose derivatives were isolated from *Pseudomonas diminuta* in 1970 (Shaw et al., 1970). Recently, an unprecedented, polyunsaturated fatty acid-rich phosphatidylmonogalactosyldiacylglycerol (PGDG) was isolated from the marine diatom *Thalassiosira weissflogii* (Manzo et al., 2019). PGDG exhibits immunostimulatory activity in human dendritic cells. To explore the mechanism of action, analogs with saturated lipids were synthesized, and Toll-like receptor-4 (TLR-4) agonist activity was demonstrated to underlie the antigen-specific T-cell activation of this class of molecules in human and mouse dendritic cells.

## 5 Conclusion and outlook

The identification and functional analysis of rare glycolipids including glycero-glycophospholipids are becoming increasingly important, not only because of their structural interest but also because of their unique biological activities, such as immunostimulatory activity and involvement in translocation of various proteins. The isolation of rare glycolipids and determination of their structures are often difficult because they are trace components of membranes and exhibit amphiphilic physicochemical properties. Mass spectrometry is typically used for glycan identification. For example, characterization of gangliosides using LC-MS is well-established. However, authentic samples are necessary for the confirmation since MS gives only molecular formula and cannot distinguish stereoisomers. Consequently, regarding novel glycolipids, isolation, derivatization, and NMR analyses are indispensable for determining the type of sugar, position of modification, and stereoconfiguration of glycerol. Purification methods, such as HPLC and liquid–liquid partition chromatography, would be useful although separation of amphiphilic compounds might require successive manipulations and complex mixture of solvents.

For biosynthetic supply and structural modification of glycolipids, molecular biological approaches are limited because glycolipids are not directly produced by the translation of genes. Chemical synthesis of the glycolipids also includes challenging issues such as the stereocontrol of glycosylation and the multistep protecting/deprotecting reactions. Nevertheless, we consider that chemical synthesis is a powerful tool for the investigation into their functions because it can supply sufficient quantities of structurally defined molecules. By conducting studies on structure-activity relationships, the detailed mechanism of the activity would be elucidated at the atomic level, which may lead to therapeutic applications. Furthermore, chemical biology using probes with detectable and/or reactive functionalities can uncover novel biological actions associated with these rare glycolipids and their target molecules.
